# Depressive Symptoms, Anxiety Disorder, and Suicide Risk During the COVID-19 Pandemic

**DOI:** 10.3389/fpsyg.2020.572699

**Published:** 2020-12-15

**Authors:** Aurel Pera

**Affiliations:** Department of Teacher Training, University of Craiova, Craiova, Romania

**Keywords:** depression, anxiety, suicide, symptom, COVID-19

## Abstract

This study reviews the existing literature on psychiatric interventions for individuals affected by the COVID-19 epidemic. My article cumulates previous research on how extreme stressors associated with COVID-19 may aggravate or cause psychiatric problems. The unpredictability of the COVID-19 epidemic progression may result in significant psychological pressure on vulnerable populations. Persons with psychiatric illnesses may experience worsening symptoms or may develop an altered mental state related to an increased suicide risk. The inspected findings prove that psychological intervention measures for patients affected by the epidemic should be designed and personalized adequately. Preventive measures seek to decrease infection rates and cut down the risk of the public healthcare system to eventually be overburdened. Throughout the COVID-19 crisis, people with psychiatric illnesses may confront a decrease in mental health services. As limitations in the current review, by focusing only on articles published in journals indexed in Web of Science, Scopus, and ProQuest, I inevitably disregarded other valuable sources. Subsequent research directions should clarify the effectiveness of online mental health services in providing remote psychiatric interventions to individuals affected by the COVID-19 epidemic.

## Introduction

COVID-19 has placed a significant strain on health systems on a large scale (Kelly, 2020b). There may be a prevalent deterioration of mental health in the affected people (Sønderskov et al., 2020). Being confined in self-isolation or quarantined may have short- to long-term adverse consequences on the mental health of affected individuals (Mukhtar, 2020). Emotional, physical, and mental fatigue may occur as a result of immoderate and persistent stress (Zhang et al., 2020a). In September 2020, I undertook a systematic review of Web of Science, Scopus, and ProQuest, employing Preferred Reporting Items for Systematic Reviews and Meta-analysis (PRISMA) guidelines. The inclusion criteria were (i) publication date: 2020 only, (ii) original empirical research, review articles, and editorial materials having non-theoretical content, (iii) written in English, and (iv) covering “COVID-19,” “depressive symptoms,” “anxiety disorder,” and “suicide risk” as search terms. I excluded from the review (i) books, (ii) proceedings papers, and (iii) theoretical comments. I have employed the Systematic Review Data Repository (SRDR), a powerful tool for the extraction, handling, and inspection of data for the systematic review. As I focused on research published exclusively this year, only 193 various types of articles met the eligibility criteria. By removing those whose results were inconclusive, unconfirmed by replication/retracted, too general, or having similar titles, I selected 68, mainly empirical, sources (Table 1).

**TABLE 1:** Topics and types of papers identified and selected.

**Topic**	**Identified**	**Selected**
COVID-19	193	68
depressive symptoms	124	61
anxiety disorder	84	56
suicide risk	75	45
**Type of Paper**		
original empirical research	103	41
review article	6	1
editorial materials	44	26
books	2	0
proceedings papers	2	0
theoretical comments	36	0

*Source: Processed by the author. Some topics overlap.*

## Health-Risk COVID-19 Behaviors Associated With Depressive Symptoms

Adequate conformity to spatial distancing in addition to home confinement, self-isolation, and quarantine may have the repercussion of social disconnection with adverse effects for psychological wellbeing (Mesa Vieira et al., 2020). The mandatory and beneficent quarantine has led to an unwitting outcome of escalated stress in old individuals dwelling in nursing homes who have a high risk of contracting COVID-19, in conjunction with increased morbidity and mortality (Padala et al., 2020). Low education level, job loss and unemployment stress, post-traumatic stress disorder symptoms, and adverse coping styles are the main determinants of mental health during the COVID-19 pandemic (Liang et al., 2020).

Fiorillo and Gorwood (2020) insist that persons who have been in direct or indirect contact with COVID-19 infected patients, who are easily affected by biological or psychosocial stressors (e.g., individuals having mental health issues), having a significant level of exposure (e.g., frontline healthcare providers), or following COVID-19 pandemic-related news (Popescu Ljungholm and Olah, 2020) provided by mainstream journalism organizations or social media platforms (Bratu, 2020a,b; Lăzăroiu et al., 2020; Rommer et al., 2020; Sheares et al., 2020) are at risk of mental health and psychosocial effects. Given that extreme stressors may aggravate or cause psychiatric problems, there will be an intensification of mental health issues, behavioral perturbations, and substance use disorders. Duan and Zhu (2020) assert that with disease evolution, clinical symptoms come to be critical and psychological issues in COVID-19 infected patients will aggravate, and thus psychological intervention measures for patients affected by the epidemic should be designed and personalized adequately.

Cao et al. (2020) show that the COVID-19 epidemic has generated excruciating psychological pressure related to the risk of passing away from infection. Having the loved ones infected with COVID-19 is an independent risk determinant for long-endured fear. Cheung et al. (2020) remark that the swiftly increasing volume of COVID-19 infected patients has been experiencing persistent fear and limitations of their current activities. Rigorous preventive measures (e.g., social distancing and extended quarantine) may adversely affect physical and psychological wellbeing, inter-familial connections and socioemotional support networks of the general population (Mircică, 2020), possibly exacerbating psychiatric morbidity, while seeking to decrease infection rates and cut down the risk of the public healthcare system to eventually be overburdened. Rajkumar (2020) writes that subsyndromal mental health issues constitute a typical reaction to the COVID-19 pandemic: symptoms of anxiety and depression in addition to self-reported stress are prevalent psychological responses and may be related to disturbed sleep.

Druss (2020) says that individuals having severe mental illnesses along with the public mental healthcare system pivotal in attending to them are at significant risk. Such patients need timely, precise information concerning approaches for diminishing risk, while discerning when to ask for COVID-19 medical treatment, and, if they are employed, they may not have a break from work and may fall short of adequate insurance coverage to secure COVID-19 testing or medical treatment. Undeveloped social networks may restrict chances to gain support from the loved ones should people having severe mental illnesses get infected with COVID-19. Apprehension may both intensify and be magnified by prevalent anxiety disorders and depressive symptoms. Shigemura et al. (2020) reveal that apprehension of the unknown during the COVID-19 crisis increases anxiety and depression levels in healthy persons and in individuals having preexisting mental health conditions, emotional responses comprising intense psychosocial distress and insecurity. Negative societal behaviors related to COVID-19 are mainly triggered by panic and misrepresented perceptions of risk.

DePierro et al. (2020) observe that the COVID-19 pandemic may give rise to high rates of post-traumatic stress disorder, depression, and substance misuse among affected people. Most individuals from underserved groups may confront chronic mental health effects by lacking the ability to access first-rate healthcare services. Mental health monitoring, timely detection of persons at risk, and medical treatment notwithstanding financial constraints are pivotal for reducing chronic distress. By harnessing cutting-edge technological devices (Nica et al., 2020; Pridmore et al., 2020; Taylor, 2020; Wright and Birtus, 2020), mental health support services can be adopted adequately by use of video telehealth platforms. Huang and Zhao (2020) hold that young persons, individuals spending excessive time mulling over the COVID-19 outbreak, and frontline healthcare providers are at significant risk of mental illness (Allen and Cug, 2020), possibly developing anxiety symptoms. The unpredictability of the COVID-19 epidemic progression may result in significant psychological pressure on vulnerable populations. Age and time spent by obsessing over COVID-19 are possible risk determinants for the psychological issues of the affected people who may have depressive symptoms and sleep problems.

On Holmes et al. (2020)’s view, COVID-19 may affect the brain or generate immune reactions that have further detrimental consequences on infected patients’ brain function and mental health, leading to harmful behaviors. The COVID-19 lockdown and social isolation may have mental health consequences for vulnerable groups, that is persons with underlying mental or physical health disorders or who show a tendency to be psychologically unwell. Psychological processes and social effects (e.g., brain function, cognition, emotion, and behavior) associated with COVID-19 (Clark, 2020a,b) may affect mental health of certain individuals who are on risk of anxiety, depression, stress, and self-inflicted violence. Remote work and job loss, together with social and physical distancing have unexpectedly discontinued numerous social prospects relevant to physical and mental health.

The COVID-19 epidemic has generated extreme stressors that may aggravate or cause psychiatric problems, affecting the brain or generating immune reactions. Emotional responses comprise intense psychosocial distress and insecurity. Such patients need timely, precise information concerning approaches for diminishing risk.

## COVID-19 Risk Factors Related to Emotional and Anxiety Disorders

COVID-19 pandemic-related, compulsory self-isolation, home confinement, and quarantine are related to poor psychological and physical health (Balanzá-Martínez et al., 2020). Taking into account the COVID-19 infection and deficiencies in personal protective equipment, direct access to patients is restricted excepting for the frontline health care providers who are trained to supply adequate medical care (Mohindra et al., 2020). Psychological interventions and social support provided to vulnerable people to handle panic, anxiety, and confinement during the COVID-19 pandemic may be efficient by use of mobile health platforms (Sim et al., 2020).

Torales et al. (2020) stress that, during COVID-19 pandemic, individuals’ emotional reactions tend to comprise extreme fear and confusion (Rommer, 2020; Thompson, 2020), while adverse social behaviors are typically activated by anxiety and misrepresented perceptions of risk. Psychosocial stressors are mainly associated with panic (Breillat and Birtus, 2020) generated by isolation or quarantine and mental instability among the affected individuals. Venkatesh and Edirappuli (2020) suggest that individuals with underlying mental illness may be affected by restricting their interpersonal relations that are pivotal to their mental healthcare, in addition to diminished access to possibly delayed psychiatric services. Personal care workers can provide specialized support (Moore and Kolencik, 2020; Sheares, 2020) by remotely monitoring individuals at risk of mental illness and vulnerable populations. Serafini et al. (2020) note that inequities and intolerance as regards to marginalized people (e.g., aged individuals having mental disorders) may be typically increased in situations of social distress, panic, irritation, and apprehension.

Lima et al. (2020) put it that primary care providers working in critical and intensive care units and hospitals should receive specialized training for supplying mental healthcare. In hopes of better handling the pressing psychological issues of individuals affected by the COVID-19 crisis, psychological crisis interventions can be enhanced by harnessing Internet technology (Lyons and Lăzăroiu, 2020), as particularly older persons having psychiatric conditions may endure additional distress. Xiang et al. (2020b) emphasize that psychiatric patients constitute an extremely vulnerable population to get infected with COVID-19, but the shortage of adequate medical procedures, insufficient mental health resources, and unsatisfactory training may prevent mental health professionals from supplying mental health services in hospitals and isolation infectious units. Assimilating mental health crisis interventions coherently in the prevalent implementation of disease prevention and treatment may curb the risk of COVID-19-related detrimental psychological outcomes.

Roy et al. (2020) state that the stress and worries in society are disturbing each person to unequal extents. There are amplified anxieties and concerns among the general population about acquiring the COVID-19 infection. Individuals have significant perceived needs to handle their mental health issues, reporting fearfulness, discomfort, and obsession about contracting such a life-threatening infectious disease, in addition to sleep disturbances. Zhang et al. (2020c) maintain that, for COVID-19 infected patients with psychiatric disorders, antiviral drugs should be used together with psychotropic ones, as otherwise they can experience deterioration of their mental illness. Due to stressor events, symptoms such as anxiety, nervousness, and insomnia may occur in such patients. Kavoor (2020) posits that deterioration of severe mental disorders associated with COVID-19 may bring about unsatisfactory hygiene, difficulties in practicing preventive strategies, dearth of prompt reporting or pursuing medical attention, and limitations in conforming to required treatment.

On Kelly (2020a)’s reading, infectious disease outbreaks such as COVID-19 bring about mental health conditions (e.g., psychiatric disorders), that is the illness brought about by the virus itself (typically self-limiting but lethal, particularly in the ill-protected people, the old individuals, and the persons having preexistent health conditions) and the fear, apprehension, and psychological issues (Dobson-Lohman and Potcovaru, 2020) related to the pandemic. Vulnerable populations (e.g., homeless persons, individuals having infirmities, chronically ill people, etc.) are at particular risk to have summative risk determinants (e.g., unsatisfactory physical and psychological health, reduced access to services, inadequate control over their ordinary activities, etc.). People with mental illness are less probable to access public health advice about COVID-19, more expected to contract the virus, and less plausible to receive expeditious adequate diagnostic and treatment services.

Moghanibashi-Mansourieh (2020) indicates that individuals’ anxious responses may lead to socially disruptive behaviors such as mass panic buying. The rampancy of mental health disorders, particularly anxiety, diminishes affected persons’ resiliency against the COVID-19 infection. Xiang et al. (2020a) find that confirmed or suspected COVID-19 patients may experience uncertainty and apprehension as regards the repercussions of acquiring a potentially deadly infection (Dawson and Potcovaru, 2020; Mihăilă and Martin, 2020; Popescu Ljungholm, 2020), while individuals in home confinement and self-isolation may confront frustration, sadness, and annoyance. Compulsory contact tracing and extended quarantine, which constitute important public health reactions to the COVID-19 outbreak, may heighten patients’ anxiety and remorse as regards the consequences of contagion and stigma on their loved ones.

Infectious disease outbreaks such as COVID-19 bring about mental health conditions as individuals’ emotional reactions tend to comprise extreme fear and confusion, while their anxious responses may lead to socially disruptive behaviors. Symptoms such as anxiety, nervousness, and insomnia may occur in COVID-19 infected patients with psychiatric disorders.

## COVID-19-Related Suicidal Ideation and Behavior

The level of risk of COVID-19 spread among persons having a severe mental illness may be more significant than that in the general population, as a consequence of constant unhealthy behaviors and standards of living (Starace and Ferrara, 2020). Individuals who stopped working as a result of the COVID-19 pandemic may experience poor mental and physical health conditions in addition to psychological distress (Zhang et al., 2020b).

As Ahmed et al. (2020) put it, social isolation and economic downsides on a large scale has led to serious psychological troubles for a lot of individuals, configuring severe neurological symptoms of COVID-19 infection. Confirmed patients are afraid of being abandoned by society and in addition to the remorse of having infecting others the outcome may be mental disturbance. The incidence of psychological issues is considerably higher among persons whose colleagues, friends or family members got infected or passed away from COVID-19. Yao et al. (2020) show that epidemics do not exert influence on all populations uniformly and imbalances can facilitate the transmission of infections. Unawareness of the distinctive effect of the epidemic on people with mental health disorders may impede any purposes to slow down increased escalation of COVID-19, while amplifying current health inequalities. Such patients may be more considerably affected by the emotional reactions generated by the COVID-19 epidemic, giving rise to deterioration of a mental health condition due to significant vulnerability to psychological distress in contrast to the general population.

Hao et al. (2020) demonstrate that comprehending the psychological consequences on patients with mental disorders throughout the COVID-19 pandemic may clarify how to set up an efficient immunopsychiatry service. Determinants to deteriorating mental health are possible postponement in supplying psychotropic medications, deficiency in access to primary care and outpatient emergency rooms, significant financial hurdles, anxiety of getting infected with COVID-19 (Hughes, 2020), prolonged interval of confinement and penurious living conditions caused by paucity of supplies. Such abrupt, unexpected changes may result in feelings of anguish and heightened suicidal ideation among people with psychiatric illnesses. Nearly all the stabilized psychiatric patients need to receive out-hospital treatment to diminish the risk of infection. Immunopsychiatry services should provide point-of-care test for COVID-19 detection, while negative results may offer moral support to persons with mental disability.

Gunnell et al. (2020) observe that the spread of COVID-19 pandemic may trigger distress, leaving affected individuals exposed to mental health disorders (Scott et al., 2020) and suicidal ideation and behavior. Severe mental health issues may be experienced by the healthy population and by persons having significant levels of vulnerability to COVID-19 illness (e.g., primary care providers and individuals who have contracted the virus). Suicide risk may be amplified due to stigma toward infected patients and their family members. Persons with psychiatric illnesses may experience worsening symptoms or may develop an altered mental state related to an increased suicide risk (e.g., anxiety, depression, and post-traumatic stress).

Reger et al. (2020) state that exceptional public health undertakings to regulate the transmission of COVID-19 will decrease the rate of further infections, but the likelihood of detrimental consequences on suicide risk is considerable. Social relationships are pivotal in suicide prevention as self-isolation and lack of companions may exacerbate suicide risk. Diminished access to mental healthcare may adversely impact patients having suicidal ideation. Aggravated physical health issues may escalate risk behavior for old patients, in whom health issues are related to deliberately killing themselves. Wang et al. (2020) point out that prolonged lockdown may have severe detrimental consequences on mental health. The persistent link between physical symptoms and the psychological effect of COVID-19 indicates the necessity of developing a swift diagnostic test having extended availability to attenuate the psychological repercussions and psychiatric symptoms endured by affected people.

On Pfefferbaum and North (2020)’s account, ambiguous prognoses, imminent drastic scarcities of resources for COVID-19 testing and treatment and for ensuring the safety of frontline healthcare providers from infection, enforcement of unusual public health actions that interfere with personal human rights, significant and increasing financial losses, and contradictory directives from governing bodies and medical experts (Lăzăroiu and Adams, 2020) constitute main stressors resulting in pervasive emotional distress and heightened risk for psychiatric disorders. Individuals who get infected with COVID-19, people at increased risk to contract the disease, and persons having preexisting psychiatric or substance use issues may develop adverse psychosocial reactions.

People with mental health disorders may be more considerably affected by the emotional reactions generated by the COVID-19 epidemic. Affected individuals are exposed to mental health disorders and suicidal ideation and behavior, resulting in pervasive emotional distress and heightened risk for psychiatric disorders. Social relationships are pivotal in suicide prevention.

## Conclusion

The swift spread of COVID-19 and important death rate may aggravate the risk of mental health issues and intensify current psychiatric symptoms, damaging their proper functioning and cognition to a greater extent (Yang et al., 2020). The conclusions drawn from the above analyses indicate the differential psychiatric distress of COVID-19 affected populations. Psychological processes and social effects associated with COVID-19 may affect mental health of certain individuals who are on risk of anxiety, depression, stress, and self-inflicted violence. Most individuals from underserved groups may confront chronic mental health effects by lacking the ability to access first-rate healthcare services. As limitations in the current review, by focusing only on articles published in journals indexed in Web of Science, Scopus, and ProQuest, I inevitably disregarded other valuable sources. Subsequent research directions should clarify the effectiveness of online mental health services in providing remote psychiatric interventions to individuals affected by the COVID-19 epidemic.

## Author Contributions

The author confirms being the sole contributor of this work and has approved it for publication.

## Conflict of Interest

The author declares that the research was conducted in the absence of any commercial or financial relationships that could be construed as a potential conflict of interest.

## References

[B1] Ahmed M. Z. Ahmed O. Aibao Z. Hanbin S. Siyu L. Ahmad A. (2020). Epidemic of COVID-19 in China and associated psychological problems. *Asian J. Psychiatr.* 51:102092. 10.1016/j.ajp.2020.102092 32315963 PMC7194662

[B2] Allen M. Cug J. (2020). Demoralization, fear, and burnout associated with being a COVID-19 frontline healthcare worker. *Psychosociol. Iss. Hum. Resour. Manage.* 8 43–48. 10.22381/PIHRM8120207

[B3] Balanzá-Martínez V. Atienza-Carbonell B. Kapczinski F. De Boni R. B. (2020). Lifestyle behaviours during the COVID−19 – time to connect. *Acta Psychiatr. Scand.* 141 399–400. 10.1111/acps.13177 32324252 PMC7264786

[B4] Bratu S. (2020a). Threat perceptions of COVID-19 pandemic: News discernment, media exaggeration, and misleading information. *Anal. Metaphys.* 19 38–44. 10.22381/AM1920203

[B5] Bratu S. (2020b). The fake news sociology of COVID-19 pandemic fear: Dangerously inaccurate beliefs, emotional contagion, and conspiracy ideation. *Linguist. Philosoph. Investig.* 19 128–134. 10.22381/LPI19202010

[B6] Breillat R. Birtus M. (2020). Is the COVID-19 outbreak severely affecting the psychological well-being of frontline respiratory and intensive care physicians and nurses? *Psychosociol. Iss. Hum. Resour. Manage.* 8 49–54. 10.22381/PIHRM8120208

[B7] Cao W. Fang Z. Hou G. Han M. Xu X. Dong J. (2020). The psychological impact of the COVID-19 epidemic on college students in China. *Psychiatr. Res.* 287:112934 10.1016/j.psychres.2020.112934 PMC7102633 32229390

[B8] Cheung T. Fong T. K. Bressington D. (2020). COVID−19 under the SARS cloud: Mental health nursing during the pandemic in Hong Kong. *J. Psychiatr. Ment. Health Nurs.* 2020:10.1111/jm.12639 10.1111/jpm.12639 PMC7264671 32311811

[B9] Clark A. (2020a). Can Internet of Medical Things improve capabilities for COVID-19 treatment and reduce transmission of the disease? *Am. J. Med. Res.* 7 57–63. 10.22381/AJMR7220208

[B10] Clark A. (2020b). COVID-19-related misinformation: Fabricated and unverified content on social media. *Anal. Metaphys.* 19 87–93. 10.22381/AM19202010

[B11] Dawson J. Potcovaru A.-M. (2020). Are female–male imbalances in COVID-19 mortality rates driven mainly by innate sex differences? *J. Res. Gender Stud.* 10 45–51. 10.22381/JRGS10120203

[B12] DePierro J. Lowe S. Katz C. (2020). Lessons learned from 9/11: Mental health perspectives on the COVID-19 pandemic. *Psychiatr. Res.* 288:113024. 10.1016/j.psychres.2020.113024 32315874 PMC7158831

[B13] Dobson-Lohman E. Potcovaru A.-M. (2020). Fake news content shaping the COVID-19 pandemic fear: Virus anxiety, emotional contagion, and responsible media reporting. *Anal. Metaphys.* 19 94–100. 10.22381/AM19202011

[B14] Druss B. G. (2020). Addressing the COVID-19 pandemic in populations with serious mental illness. *JAMA Psychiatr.* 77 891–892. 10.1001/jamapsychiatry.2020.0894 32242888

[B15] Duan L. Zhu G. (2020). Psychological interventions for people affected by the COVID-19 epidemic. *Lancet Psychiatr.* 7 300–302. 10.1016/S2215-0366(20)30073-0 PMC7128328 32085840

[B16] Fiorillo A. Gorwood P. (2020). The consequences of the COVID-19 pandemic on mental health and implications for clinical practice. *Eur. Psychiatr.* 63:e32. 10.1192/j.eurpsy.2020.35 32234102 PMC7156565

[B17] Gunnell D. Appleby L. Arensman E. Hawton K. John A. Kapur N. (2020). Suicide risk and prevention during the COVID-19 pandemic. *Lancet Psychiatr.* 7 468–471. 10.1016/S2215-0366(20)30171-1 PMC7173821 32330430

[B18] Hao F. Tan W. Jiang L. Zhang L. Zhao X. Zou Y. (2020). Do psychiatric patients experience more psychiatric symptoms during COVID-19 pandemic and lockdown? A case-control study with service and research implications for immunopsychiatry. *Brain Behav. Immun.* 87 100–106. 10.1016/j.bbi.2020.04.069 32353518 PMC7184991

[B19] Holmes E. A. O’Connor R. C. Perry V. H. Tracey I. Wessely S. Arseneault L. (2020). Multidisciplinary research priorities for the COVID-19 pandemic: a call for action for mental health science. *Lancet Psychiatr.* 7 547–560. 10.1016/S2215-0366(20)30168-1 PMC7159850 32304649

[B20] Huang Y. Zhao N. (2020). Generalized anxiety disorder, depressive symptoms and sleep quality during COVID-19 outbreak in China: a web-based cross-sectional survey. *Psychiatr. Res.* 288:112954. 10.1016/j.psychres.2020.112954 32325383 PMC7152913

[B21] Hughes A. (2020). Artificial intelligence-enabled healthcare delivery and real-time medical data analytics in monitoring, detection, and prevention of COVID-19. *Am. J. Medic. Res.* 7 50–56. 10.22381/AJMR7220207

[B22] Kavoor A. R. (2020). COVID-19 in people with mental illness: Challenges and vulnerabilities. *Asian J. Psychiatr.* 51:102051. 10.1016/j.ajp.2020.102051 32298968 PMC7139245

[B23] Kelly B. D. (2020a). Covid-19 (Coronavirus): Challenges for psychiatry. *Br. J. Psychiatr.* 217 352–353. 10.1192/bjp.2020.86 32293555 PMC7205546

[B24] Kelly B. D. (2020b). Emergency mental health legislation in response to the Covid-19 (Coronavirus) pandemic in Ireland: Urgency, necessity and proportionality. *Int. J. Law Psychiatr.* 70:101564. 10.1016/j.ijlp.2020.101564 32482306 PMC7174175

[B25] Lăzăroiu G. Adams C. (2020). Viral panic and contagious fear in scary times: The proliferation of COVID-19 misinformation and fake news. *Anal. Metaphys.* 19 80–86. 10.22381/AM1920209

[B26] Lăzăroiu G. Horak J. Valaskova K. (2020). Scaring ourselves to death in the time of COVID-19: Pandemic awareness, virus anxiety, and contagious fear. *Linguist. Philosoph. Investig.* 19 114–120. 10.22381/LPI1920208

[B27] Liang L. Ren H. Cao R. Hu Y. Qin Z. Li C. (2020). The effect of COVID-19 on youth mental health. *Psychiatr. Quart.* 91 841–852. 10.1007/s11126-020-09744-3 PMC7173777 32319041

[B28] Lima C. K. T. Carvalho P. M. M. Lima I. A. A. S. Nunes J. V. A. O. Saraiva J. S. de Souza R. I. da Silva C. G. L. (2020). The emotional impact of Coronavirus 2019-nCoV (new Coronavirus disease). *Psychiatr. Res.* 287:112915. 10.1016/j.psychres.2020.112915 32199182 PMC7195292

[B29] Lyons N. Lăzăroiu G. (2020). Addressing the COVID-19 crisis by harnessing Internet of Things sensors and machine learning algorithms in data-driven smart sustainable cities. *Geopolit. His. Int. Relat.* 12 65–71. 10.22381/GHIR12220209

[B30] Mesa Vieira C. Franco O. H. Gómez Restrepo C. Abel T. (2020). COVID-19: The forgotten priorities of the pandemic. *Maturitas* 136 38–41. 10.1016/j.maturitas.2020.04.004 32386664 PMC7195319

[B31] Mihăilă R. Martin A. (2020). The impact of sex and gender on the incidence and case fatality of COVID-19 infection. *J. Res. Gender Stud.* 10 38–44. 10.22381/JRGS10120202

[B32] Mircică N. (2020). Restoring public trust in digital platform operations: Machine learning algorithmic structuring of social media content. *Rev. Contemp. Philos.* 19 85–91. 10.22381/RCP1920209

[B33] Moghanibashi-Mansourieh A. (2020). Assessing the anxiety level of Iranian general population during COVID-19 outbreak. *Asian J. Psychiatr.* 51:102076. 10.1016/j.ajp.2020.102076 32334409 PMC7165107

[B34] Mohindra R. Ravaki R. Suri V. Bhalla A. Singh S. M. (2020). Issues relevant to mental health promotion in frontline health care providers managing quarantined/isolated COVID19 patients. *Asian J. Psychiatr.* 51:102084. 10.1016/j.ajp.2020.102084 32289728 PMC7138416

[B35] Moore C. Kolencik J. (2020). Acute depression, extreme anxiety, and prolonged stress among COVID-19 frontline healthcare workers. *Psychosociol. Iss. Hum. Resour. Manage.* 8 55–60. 10.22381/PIHRM8120209

[B36] Mukhtar S. (2020). Mental health and psychosocial aspects of coronavirus outbreak in Pakistan: Psychological intervention for public mental health crisis. *Asian J. Psychiatr.* 51:102069. 10.1016/j.ajp.2020.102069 32344331 PMC7161472

[B37] Nica E. Kliestik T. Sabie O.-M. Ioanei M.-L. (2020). Socio-affective technologies for psychological health: Emotional artificial intelligence in empathetic robots. *Am. J. Medic. Res.* 7 9–14. 10.22381/AJMR7220201

[B38] Padala S. P. Jendro A. M. Orr L. C. (2020). Facetime to reduce behavioral problems in a nursing home resident with Alzheimer’s dementia during COVID-19. *Psychiatr. Res.* 288:113028. 10.1016/j.psychres.2020.113028 32361337 PMC7177115

[B39] Pfefferbaum B. North C. S. (2020). Mental health and the Covid-19 pandemic. *N. Engl. J. Med.* 383 510–512. 10.1056/NEJMp2008017 32283003

[B40] Popescu Ljungholm D. (2020). COVID-19 vaccine safety regulation: Globalized infectious diseases, health literacy, and anti-immunization sentiment. *Anal. Metaphys.* 19 31–37. 10.22381/AM1920202

[B41] Popescu Ljungholm D. Olah M. L. (2020). Regulating fake news content during COVID-19 pandemic: Evidence-based reality, trustworthy sources, and responsible media reporting. *Rev. Contemp. Philos.* 19 43–49. 10.22381/RCP1920203

[B42] Pridmore S. Erger S. Rybak M. Pridmore W. (2020). The Mood Log – A tool for clustered maintenance TMS. *Am. J. Med. Res.* 7 15–21. 10.22381/AJMR7220202

[B43] Rajkumar R. P. (2020). COVID-19 and mental health: A review of the existing literature. *Asian J. Psychiatr.* 52:102066. 10.1016/j.ajp.2020.102066 32302935 PMC7151415

[B44] Reger M. A. Stanley I. H. Joiner T. E. (2020). Suicide mortality and coronavirus disease 2019 – A perfect storm? *JAMA Psychiatr.* Preprint. 10.1001/jamapsychiatry.2020.1060 32275300

[B45] Rommer D. (2020). The psychological ill-health of frontline medical staff working with COVID-19 patients: Burnout, anxiety, and post-traumatic stress disorder. *Psychosociol. Iss. Hum. Res. Manage.* 8 13–18. 10.22381/PIHRM8120202

[B46] Rommer D. Majerova J. Machova V. (2020). Repeated COVID-19 pandemic-related media consumption: Minimizing sharing of nonsensical misinformation through health literacy and critical thinking. *Linguist. Philosop. Investig.* 19 107–113. 10.22381/LPI1920207

[B47] Roy D. Tripathy S. Kar S. K. Sharma N. Verma S. K. Kaushal V. (2020). Study of knowledge, attitude, anxiety & perceived mental healthcare need in Indian population during COVID-19 pandemic. *Asian J. Psychiatr.* 51:102083 10.1016/j.ajp.2020.102083 PMC7139237 32283510

[B48] Scott R. Poliak M. Vrbka J. Nica E. (2020). COVID-19 response and recovery in smart sustainable city governance and management: Data-driven Internet of Things systems and machine learning-based analytics. *Geopolit. His. Int. Relat.* 12 16–22. 10.22381/GHIR12220202

[B49] Serafini G. Bondi E. Locatelli C. Amore M. (2020). Aged patients with mental disorders in the COVID-19 era: The experience of Northern Italy. *Am. J. Geriatr. Psychiatr.* 28 794–795. 10.1016/j.jagp.2020.04.015 32360137 PMC7175843

[B50] Sheares G. (2020). Psychological well-being of COVID-19 medical staff: Depression, distress, and anxiety in frontline healthcare workers. *Psychosociol. Iss. Hum. Resour. Manage.* 8 37–42. 10.22381/PIHRM8120206

[B51] Sheares G. Miklencicova R. Grupac M. (2020). The viral power of fake news: Subjective social insecurity, COVID-19 damaging misinformation, and baseless conspiracy theories. *Linguist. Philosop. Investig.* 19 121–127. 10.22381/LPI1920209

[B52] Shigemura J. Ursano R. J. Morganstein J. C. Kurosawa M. Benedek D. M. (2020). Public responses to the novel 2019 coronavirus (2019−nCoV) in Japan: Mental health consequences and target populations. *Psychiatr. Clin. Neurosci.* 74 281–282. 10.1111/pcn.12988 PMC7168047 32034840

[B53] Sim K. Chua H. C. Vieta E. Fernandez G. (2020). The anatomy of panic buying related to the current COVID-19 pandemic. *Psychiatr. Res.* 288:113015. 10.1016/j.psychres.2020.113015 32315887 PMC7158779

[B54] Sønderskov K. M. Dinesen P. T. Santini Z. I. Østergaard S. D. (2020). The depressive state of Denmark during the COVID-19 pandemic. *Acta Neuropsychiatr.* 32 226–228. 10.1017/neu.2020.15 32319879 PMC7176490

[B55] Starace F. Ferrara M. (2020). COVID-19 disease emergency operational instructions for Mental Health Departments issued by the Italian Society of Epidemiological Psychiatry. *Epidemiol. Psychiatr. Sci.* 29:e116 10.1017/S2045796020000372 PMC7163186 32228737

[B56] Taylor L. (2020). Healthcare body sensor networks and smart wearable devices in Internet of Medical Things. *Am. J. Med. Res.* 7 67–73. 10.22381/AJMR71202010

[B57] Thompson D. (2020). Psychological trauma symptoms and mental conditions of medical staff during the COVID-19 pandemic: Severe stress, elevated anxiety, and clinically significant depression. *Psychosociol. Iss. Hum. Resour. Manage.* 8 25–30. 10.22381/PIHRM8120204

[B58] Torales J. O’Higgins M. Castaldelli-Maia J. M. Ventriglio A. (2020). The outbreak of COVID-19 coronavirus and its impact on global mental health. *Int. J. Soc. Psychiatr.* 66 317–320. 10.1177/0020764020915212 32233719

[B59] Venkatesh A. Edirappuli S. (2020). Social distancing in covid-19: What are the mental health implications? *BMJ* 369:m1379 10.1136/bmj.m1379 32253182

[B60] Wang C. Pan R. Wan X. Tan Y. Xu L. McIntyre R. S. (2020). A longitudinal study on the mental health of general population during the COVID-19 epidemic in *China*. *Brain Behav. Immun.* 87 40–48. 10.1016/j.bbi.2020.04.028 32298802 PMC7153528

[B61] Wright S. Birtus M. (2020). Real-time medical data analytics in Internet of Things-based smart healthcare systems. *Am. J. Med. Res.* 7 61–66. 10.22381/AJMR7120209

[B62] Xiang Y.-T. Jin Y. Cheung T. (2020a). Joint international collaboration to combat mental health challenges during the coronavirus disease 2019 pandemic. *JAMA Psychiatr.* 77 989–990. 10.1001/jamapsychiatry.2020.1057 32275289

[B63] Xiang Y.-T. Yang Y. Li W. Zhang L. Zhang Q. Cheung T. (2020b). Timely mental health care for the 2019 novel coronavirus outbreak is urgently needed. *Lancet Psychiatr.* 7 228–229. 10.1016/S2215-0366(20)30046-8 PMC7128153 32032543

[B64] Yang Y. Li W. Zhang Q. Zhang L. Cheung T. Xiang Y.-T. (2020). Mental health services for older adults in China during the COVID-19 outbreak. *Lancet Psychiatr.* 7:e19 10.1016/S2215-0366(20)30079-1 PMC7128970 32085843

[B65] Yao H. Chen J.-H. Xu Y.-F. (2020). Patients with mental health disorders in the COVID-19 epidemic. *Lancet Psychiatr.* 7:e21 10.1016/S2215-0366(20)30090-0 PMC7269717 32199510

[B66] Zhang K. Zhou X. Liu H. Hashimoto K. (2020c). Treatment concerns for psychiatric symptoms in COVID-19-infected patients with or without psychiatric disorders. *Br. J. Psychiatr.* 217:351. 10.1192/bjp.2020.84 32270760 PMC7190394

[B67] Zhang S. X. Huang H. Wei F. (2020a). Geographical distance to the epicenter of Covid-19 predicts the burnout of the working population: Ripple effect or typhoon eye effect? *Psychiatr. Res.* 288:112998. 10.1016/j.psychres.2020.112998 32325386 PMC7194871

[B68] Zhang S. X. Wang Y. Rauch A. Wei F. (2020b). Unprecedented disruption of lives and work: Health, distress and life satisfaction of working adults in China one month into the COVID-19 outbreak. *Psychiatr. Res.* 288:112958. 10.1016/j.psychres.2020.112958 32283450 PMC7146665

