# Supplementary material for: Depressive Symptoms, Anxiety Disorder, and Suicide Risk During the COVID-19 Pandemic
Source: Front Psychol. 2020 Dec 15;11:572699. doi: 10.3389/fpsyg.2020.572699 (PMC7769944; doi:10.3389/fpsyg.2020.572699)
Supplement: Supplementary file 1 [file Table_1.DOC]

Removed titles

| No | Title | Type | Reason |
| --- | --- | --- | --- |
| 1 | https://pubmed.ncbi.nlm.nih.gov/32194290/ | original empirical research | similar title |
| 2 | https://www.sciencedirect.com/science/article/pii/S0022395614001861 | original empirical research | retracted |
| 3 | https://www.nature.com/articles/s41598-020-58462-0 | original empirical research | retracted |
| 4 | https://www.tandfonline.com/doi/full/10.1080/14767058.2020.1763946 | original empirical research | inconclusive |
| 5 | https://www.x-mol.com/paper/1294273830262022144 | original empirical research | retracted |
| 6 | https://www.hindawi.com/journals/crips/2016/9802939/ | original empirical research | retracted |
| 7 | https://www.sciencedirect.com/science/article/pii/S1876201820305220?via%3Dihub | original empirical research | retracted |
| 8 | https://journals.plos.org/plosone/article?id=10.1371/journal.pone.0238416 | original empirical research | similar title |
| 9 | https://jamanetwork.com/journals/jamanetworkopen/fullarticle/2770146 | original empirical research | inconclusive |
| 10 | https://journals.sagepub.com/doi/full/10.1177/0020764020935488 | original empirical research | similar title |
| 11 | https://journals.plos.org/plosone/article?id=10.1371/journal.pone.0238162 | original empirical research | inconclusive |
| 12 | https://www.hindawi.com/journals/drt/2020/3158954/ | original empirical research | similar title |
| 13 | https://www.frontiersin.org/articles/10.3389/fpsyg.2020.561609/full | original empirical research | inconclusive |
| 14 | https://academic.oup.com/psychsocgerontology/advance-article/doi/10.1093/geronb/gbaa110/5890805 | original empirical research | similar title |
| 15 | https://www.thelancet.com/journals/eclinm/article/PIIS2589-5370(20)30168-1/fulltext | original empirical research | inconclusive |
| 16 | https://link.springer.com/article/10.1007/s11482-020-09851-0 | original empirical research | similar title |
| 17 | https://www.sciencedirect.com/science/article/pii/S0887618520300463 | original empirical research | inconclusive |
| 18 | https://onlinelibrary.wiley.com/doi/10.1002/brb3.1745 | original empirical research | inconclusive |
| 19 | https://psychotherapy.psychiatryonline.org/doi/10.1176/appi.psychotherapy.20200015 | original empirical research | similar title |
| 20 | https://advances.sciencemag.org/content/6/42/eabd5390 | original empirical research | inconclusive |
| 21 | https://www.frontiersin.org/articles/10.3389/fpsyg.2020.565688/full | original empirical research | inconclusive |
| 22 | https://www.mdpi.com/1660-4601/17/9/3165/htm | original empirical research | inconclusive |
| 23 | https://link.springer.com/article/10.1007/s10879-020-09467-3 | original empirical research | inconclusive |
| 24 | https://f1000research.com/articles/9-1115 | original empirical research | similar title |
| 25 | https://jamanetwork.com/journals/jamainternalmedicine/fullarticle/2769543 | original empirical research | inconclusive |
| 26 | https://www.ncbi.nlm.nih.gov/pmc/articles/PMC7335942/ | original empirical research | similar title |
| 27 | https://link.springer.com/article/10.1007/s10608-020-10143-y | original empirical research | inconclusive |
| 28 | https://www.hindawi.com/journals/drt/2020/8887727/ | original empirical research | similar title |
| 29 | https://www.sciencedirect.com/science/article/pii/S1555415520305316 | original empirical research | inconclusive |
| 30 | https://www.sciencedirect.com/science/article/pii/S016517812031252X | original empirical research | inconclusive |
| 31 | https://onlinelibrary.wiley.com/doi/full/10.1002/da.23080 | original empirical research | similar title |
| 32 | https://link.springer.com/article/10.1186/s12992-020-00587-y | original empirical research | inconclusive |
| 33 | https://www.sciencedirect.com/science/article/pii/S0191886920306462 | original empirical research | inconclusive |
| 34 | https://www.sciencedirect.com/science/article/pii/S0165032720323879 | original empirical research | inconclusive |
| 35 | https://www.ncbi.nlm.nih.gov/pmc/articles/PMC7129111/ | original empirical research | similar title |
| 36 | https://www.sciencedirect.com/science/article/pii/S1744388120302784 | original empirical research | inconclusive |
| 37 | https://link.springer.com/article/10.1007/s00406-020-01171-6 | original empirical research | inconclusive |
| 38 | https://www.karger.com/Article/FullText/510752 | original empirical research | inconclusive |
| 39 | https://onlinelibrary.wiley.com/doi/abs/10.1002/sres.2658 | original empirical research | similar title |
| 40 | https://www.ncbi.nlm.nih.gov/pmc/articles/PMC7169930/ | original empirical research | inconclusive |
| 41 | https://www.sciencedirect.com/science/article/pii/S1755296620300296 | original empirical research | inconclusive |
| 42 | https://www.sciencedirect.com/science/article/pii/S2213219820303275 | original empirical research | inconclusive |
| 43 | https://www.ncbi.nlm.nih.gov/pmc/articles/PMC7200846/ | original empirical research | similar title |
| 44 | https://www.tandfonline.com/doi/full/10.1080/02646838.2020.1786037 | original empirical research | inconclusive |
| 45 | https://www.ncbi.nlm.nih.gov/pmc/articles/PMC7342099/ | original empirical research | inconclusive |
| 46 | https://www.sciencedirect.com/science/article/pii/S016517812031996X | original empirical research | inconclusive |
| 47 | https://www.ncbi.nlm.nih.gov/pmc/articles/PMC7185265/ | original empirical research | similar title |
| 48 | https://www.sciencedirect.com/science/article/pii/S0165178120311185 | original empirical research | inconclusive |
| 49 | https://www.ncbi.nlm.nih.gov/pmc/articles/PMC7118532/ | original empirical research | inconclusive |
| 50 | https://www.sciencedirect.com/science/article/pii/S0887618520300852 | original empirical research | inconclusive |
| 51 | https://www.sciencedirect.com/science/article/pii/S0191886920305389 | original empirical research | similar title |
| 52 | https://www.ncbi.nlm.nih.gov/pmc/articles/PMC7100496/ | original empirical research | inconclusive |
| 53 | https://www.tandfonline.com/doi/full/10.1080/14616696.2020.1828975 | original empirical research | inconclusive |
| 54 | https://www.sciencedirect.com/science/article/pii/S1064748120304000 | original empirical research | inconclusive |
| 55 | https://www.ncbi.nlm.nih.gov/pmc/articles/PMC7156946/ | original empirical research | inconclusive |
| 56 | https://www.tandfonline.com/doi/full/10.1080/13548506.2020.1746817 | original empirical research | similar title |
| 57 | https://onlinelibrary.wiley.com/doi/full/10.1111/jsr.13052 | review article | inconclusive |
| 58 | https://www.nature.com/articles/s41562-020-0884-z | review article | too general |
| 59 | https://pubmed.ncbi.nlm.nih.gov/32202824/ | review article | inconclusive |
| 60 | https://academic.oup.com/schizophreniabulletin/article/46/4/752/5826166 | review article | similar title |
| 61 | https://www.jpsmjournal.com/article/S0885-3924(20)30207-4/fulltext | review article | too general |
| 62 | https://www.healio.com/nursing/journals/jgn/2020-5-46-5/%7B8085aace-e2d6-4604-ad51-aa001818b1fd%7D/public-health-and-ethics-intersect-at-new-levels-with-gerontological-nursing-in-covid-19-pandemic | editorial materials | too general |
| 63 | https://link.springer.com/article/10.1007/s40737-020-00178-5 | editorial materials | too general |
| 64 | https://onlinelibrary.wiley.com/doi/10.1111/add.15080 | editorial materials | similar title |
| 65 | https://www.tandfonline.com/doi/abs/10.1080/09540962.2020.1748855?journalCode=rpmm20 | editorial materials | too general |
| 66 | https://onlinelibrary.wiley.com/doi/10.1111/jocn.15290 | editorial materials | too general |
| 67 | https://www.cmaj.ca/content/192/13/E340 | editorial materials | similar title |
| 68 | https://pubmed.ncbi.nlm.nih.gov/32271070/ | editorial materials | similar title |
| 69 | https://www.ncbi.nlm.nih.gov/pmc/articles/PMC7161513/ | editorial materials | too general |
| 70 | https://www.ncbi.nlm.nih.gov/pmc/articles/PMC7127630/ | editorial materials | similar title |
| 71 | https://pubmed.ncbi.nlm.nih.gov/32339041/ | editorial materials | too general |
| 72 | https://www.cambridge.org/core/journals/psychological-medicine/article/mental-health-characteristics-associated-with-dysfunctional-coronavirus-anxiety/FFD3CA27D3669494A91F78DC68CCE2FA | editorial materials | too general |
| 73 | https://www.thelancet.com/journals/lanpsy/article/PIIS2215-0366(20)30307-2/fulltext | editorial materials | similar title |
| 74 | https://journals.sagepub.com/doi/full/10.1177/0706743720935646 | editorial materials | too general |
| 75 | https://onlinelibrary.wiley.com/doi/full/10.1002/da.23014?af=R | editorial materials | similar title |
| 76 | https://www.ncbi.nlm.nih.gov/pmc/articles/PMC7138159/ | editorial materials | too general |
| 77 | https://pubmed.ncbi.nlm.nih.gov/32281130/ | editorial materials | too general |
| 78 | https://robotics.sciencemag.org/content/5/40/eabb5589 | editorial materials | similar title |
| 79 | https://pubmed.ncbi.nlm.nih.gov/32266767/ | editorial materials | too general |
| 80 | Mental Health and the COVID-19 Pandemic: The Essentials [Elisa M Brietzke, MD PhD](https://www.google.ro/search?tbo=p&tbm=bks&q=inauthor:"Elisa+M+Brietzke,+MD+PhD"), [Roger S. McIntyre](https://www.google.ro/search?tbo=p&tbm=bks&q=inauthor:"Roger+S.+McIntyre"), [Rodrigo Grassi-Oliveira, MD PhD](https://www.google.ro/search?tbo=p&tbm=bks&q=inauthor:"Rodrigo+Grassi-Oliveira,+MD+PhD"), [Lin Kangguang, MD](https://www.google.ro/search?tbo=p&tbm=bks&q=inauthor:"Lin+Kangguang,+MD")  Elsevier - Health Sciences Division | books | too general |
| 81 | **Community Public Health in Policy and Practice E-Book: A Sourcebook**  edited by Sarah Cowley, Karen Whittaker  Elsevier - Health Sciences Division | books | too general |
| 82 | [Interactive videoconferencing in the redesign of a health-care quality improvement workshop for the coronavirus disease 2019 pandemic](https://apps-webofknowledge-com.am.e-nformation.ro/full_record.do?product=WOS&search_mode=GeneralSearch&qid=2&SID=F5yCF5Lr1X1e6yWuDoz&page=2&doc=13)  By: [Seow, Yee Ting](https://apps-webofknowledge-com.am.e-nformation.ro/OutboundService.do?SID=F5yCF5Lr1X1e6yWuDoz&mode=rrcAuthorRecordService&action=go&product=WOS&lang=en_US&daisIds=41740221); [Teo, Shao Chu](https://apps-webofknowledge-com.am.e-nformation.ro/OutboundService.do?SID=F5yCF5Lr1X1e6yWuDoz&mode=rrcAuthorRecordService&action=go&product=WOS&lang=en_US&daisIds=41741736); [Yap, William](https://apps-webofknowledge-com.am.e-nformation.ro/OutboundService.do?SID=F5yCF5Lr1X1e6yWuDoz&mode=rrcAuthorRecordService&action=go&product=WOS&lang=en_US&daisIds=41748384); et al.  PROCEEDINGS OF SINGAPORE HEALTHCARE     Article Number: 2010105820961795 | proceedings papers | too general |
| 83 | [Prospective planning of radiation treatment at new patient conference during the COVID-19 Pandemic: The University of Mississippi Medical Center experience.](https://apps-webofknowledge-com.am.e-nformation.ro/full_record.do?product=WOS&search_mode=GeneralSearch&qid=2&SID=F5yCF5Lr1X1e6yWuDoz&page=5&doc=49)  By: [Joseph, Sanjay](https://apps-webofknowledge-com.am.e-nformation.ro/OutboundService.do?SID=F5yCF5Lr1X1e6yWuDoz&mode=rrcAuthorRecordService&action=go&product=WOS&lang=en_US&daisIds=29701888); [Nittala, Mary](https://apps-webofknowledge-com.am.e-nformation.ro/OutboundService.do?SID=F5yCF5Lr1X1e6yWuDoz&mode=rrcAuthorRecordService&action=go&product=WOS&lang=en_US&daisIds=11220758); [Roberts, Paul](https://apps-webofknowledge-com.am.e-nformation.ro/OutboundService.do?SID=F5yCF5Lr1X1e6yWuDoz&mode=rrcAuthorRecordService&action=go&product=WOS&lang=en_US&daisIds=475104); et al.  Conference: Annual Meeting of the American-Association-for-Cancer-Research (AACR) Location: ‏ ELECTR NETWORK Date: ‏ JUL 20-22, 2020  Sponsor(s): ‏Amer Assoc Canc Res | proceedings papers | too general |
| 84 | https://www.sciencedirect.com/science/article/pii/S0887618520300827 | original empirical research | inconclusive |
| 85 | https://journals.sagepub.com/doi/full/10.1177/0020764020935488 | original empirical research | similar title |
| 86 | https://www.jmir.org/2020/8/e20328/ | original empirical research | inconclusive |
| 87 | https://onlinelibrary.wiley.com/doi/full/10.1002/brb3.1745 | original empirical research | similar title |
| 88 | https://onlinelibrary.wiley.com/doi/full/10.1002/jts.22600 | original empirical research | inconclusive |
| 89 | https://academic.oup.com/ibdjournal/advance-article/doi/10.1093/ibd/izaa261/5920974 | original empirical research | similar title |
| 90 | https://www.nature.com/articles/s41591-020-0874-8 | theoretical comments | too general |
| 91 | https://www.ncbi.nlm.nih.gov/pmc/articles/PMC7153523/ | theoretical comments | too general |
| 92 | https://www.tandfonline.com/doi/full/10.1080/17476348.2020.1787835 | theoretical comments | similar title |
| 93 | https://erj.ersjournals.com/content/56/1/2001704.abstract | theoretical comments | too general |
| 94 | https://www.sciencedirect.com/science/article/pii/S0020748920302674 | theoretical comments | similar title |
| 95 | https://www.sciencedirect.com/science/article/abs/pii/S0889159120306371 | theoretical comments | too general |
| 96 | https://www.sciencedirect.com/science/article/pii/S0165032720323855 | theoretical comments | too general |
| 97 | https://www.ncbi.nlm.nih.gov/pmc/articles/PMC7177120/ | theoretical comments | similar title |
| 98 | https://www.thelancet.com/journals/lancet/article/PIIS0140-6736(20)30755-8/fulltext | theoretical comments | too general |
| 99 | https://www.tandfonline.com/doi/full/10.1080/0145935X.2020.1835163 | theoretical comments | similar title |
| 100 | https://www.sciencedirect.com/science/article/pii/S0165178120317765 | theoretical comments | too general |
| 101 | https://obgyn.onlinelibrary.wiley.com/doi/full/10.1111/1471-0528.16431 | theoretical comments | too general |
| 102 | https://onlinelibrary.wiley.com/doi/abs/10.1111/acer.14468 | theoretical comments | similar title |
| 103 | https://academic.oup.com/jpepsy/article/45/8/839/5885276 | theoretical comments | too general |
| 104 | https://www.sciencedirect.com/science/article/pii/S0010440X20300225 | theoretical comments | similar title |
| 105 | https://www.sciencedirect.com/science/article/pii/S0889159120303913 | theoretical comments | too general |
| 106 | https://www.acpjournals.org/doi/10.7326/M20-1678 | theoretical comments | too general |
| 107 | https://www.ncbi.nlm.nih.gov/pmc/articles/PMC7118448/ | theoretical comments | similar title |
| 108 | https://www.tandfonline.com/doi/full/10.1080/08959420.2020.1759758 | theoretical comments | too general |
| 109 | https://www.thelancet.com/journals/landia/article/PIIS2213-8587(20)30154-6/fulltext | theoretical comments | too general |
| 110 | https://www.thelancet.com/journals/lancet/article/PIIS0140-6736(20)30309-3/fulltext | theoretical comments | similar title |
| 111 | https://ps.psychiatryonline.org/doi/10.1176/appi.ps.202000166 | theoretical comments | too general |
| 112 | https://journals.sagepub.com/doi/full/10.1177/2047487320916823 | theoretical comments | too general |
| 113 | https://www.ncbi.nlm.nih.gov/pmc/articles/PMC7102520/ | theoretical comments | similar title |
| 114 | https://www.ncbi.nlm.nih.gov/pmc/articles/PMC7417894/ | theoretical comments | too general |
| 115 | https://jamanetwork.com/journals/jamanetworkopen/article-abstract/2770142 | theoretical comments | similar title |
| 116 | https://link.springer.com/article/10.1007/s11077-020-09381-4 | theoretical comments | too general |
| 117 | https://www.liebertpub.com/doi/full/10.1089/tmj.2020.0068 | theoretical comments | similar title |
| 118 | https://www.ncbi.nlm.nih.gov/pmc/articles/PMC7111296/ | theoretical comments | too general |
| 119 | https://academic.oup.com/workar/article/6/4/254/5919539 | theoretical comments | too general |
| 120 | https://obgyn.onlinelibrary.wiley.com/doi/full/10.1111/1471-0528.16192 | theoretical comments | too general |
| 121 | https://academic.oup.com/qjmed/article/113/5/311/5813733 | theoretical comments | similar title |
| 122 | https://jamanetwork.com/journals/jamainternalmedicine/fullarticle/2764404 | theoretical comments | too general |
| 123 | https://psycnet.apa.org/fulltext/2020-39196-001.pdf | theoretical comments | too general |
| 124 | https://journals.sagepub.com/doi/abs/10.1177/0020764020963150?journalCode=ispa | theoretical comments | too general |
| 125 | https://psycnet.apa.org/fulltext/2020-41459-001.html | theoretical comments | too general |
